# Differential Expression of *ACTL8* Gene and Association Study of Its Variations with Growth Traits in Chinese Cattle

**DOI:** 10.3390/ani9121068

**Published:** 2019-12-02

**Authors:** Cuicui Cai, Jiawei Xu, Yongzhen Huang, Xianyong Lan, Chuzhao Lei, Xueyao Yang, Jianliang Xie, Yuhua Li, Hong Chen

**Affiliations:** 1 Shaanxi Key Laboratory of Molecular Biology for Agriculture, College of Animal Science and Technology, Northwest A&F University, Xianyang 712100, China; caicuicui3c@163.com (C.C.); xjwsci@126.com (J.X.); hyzsci@126.com (Y.H.); lanxianyong79@nwsuaf.edu.cn (X.L.); leichuzhao1118@sina.com (C.L.); 2 Guyuan Branch of Ningxia Academy of Agriculture and Forestry Sciences, Guyuan 756000, China; jolinrenee@163.com; 3 Service Center for Animal Husbandry Technology in Guyuan City, Guyuan 756000, China; nmjxjl26@163.com (J.X.); 13995003216@139.com (Y.L.)

**Keywords:** cattle, *ACTL8* gene, genetic variant, association, real-time quantitative

## Abstract

**Simple Summary:**

Marker-assisted selection has a great influence on livestock molecular breeding development. The discovery of key molecular markers that are significantly associated with body size data will accelerate molecular breeding in livestock. In this study, the cattle *ACTL8* gene is a critical candidate gene. It was found that there are multiple mutations in the *ACTL8* gene that may be used as molecular markers. Our results have shown that the mutations of the *ACTL8* gene could have important reference value in molecular breeding for beef cattle.

**Abstract:**

Mutations are heritable changes at the base level of genomic DNA. Furthermore, mutations lead to genetic polymorphisms and may alter animal growth phenotypes. Our previous study found that mutations in the bovine Actin-like protein 8 (*ACTL8*) gene may be involved in muscle growth and development. This study explored several mutations of the *ACTL8* gene and their influence on body size in Chinese beef cattle, as well as tested the tissue expression profile of the *ACTL8* gene in Qinchuan cattle at different ages. Five single nucleotide polymorphisms (SNPs) (including one synonymous mutation (c.2135552895G > A)) and two insertion/deletion polymorphisms (indels) were identified in the *ACTL8* gene from 1138 cattle by DNA-seq, RFLP and other methods. Then, the expression profile of the *ACTL8* gene in Qinchuan cattle showed that it was expressed in heart, spleen, lung, liver, muscle, and fat tissues. Moreover, the expression level of ACTL8 was increased with cattle growth (*p* < 0.01). The ACTL8 mRNA expression level in kidney and muscle tissues was the highest in the calves, while lowest in the fetal stage. Overall, we showed that the mutations could act as markers in beef molecular breeding and selection of the growth traits of cattle.

## 1. Introduction

Recently, with the selection pressure strongly increased, genetic diversity is being lost in most local and industrial cattle breeds at a worldwide level. Genetic diversity in cattle is thus highly endangered [1]. Further, the genetic diversity of beef production traits is gradually decreasing, while people are increasingly demanding beef meat quality traits [2]. It is urgent to identify and utilize the genetic diversity of traits related to bovine meat production through biotechnology [3]. The discovery and application of DNA molecular marker-assisted selection technology has promoted the process of animal genetic improvement. The continuous excavation of DNA molecular markers combined with the traditional phenotypic breeding of cattle provides great help for the identification and utilization of bovine genetic resources [4]. The model which links the molecular markers and growth traits has been used for improved genetic evaluation of beef cattle [5]. However, effective candidate genes for phenotypic traits and related DNA markers remain to be discovered.

According to previous reports, actin was discovered in muscle tissues by Straub in 1942. In muscle cells, the actin protein as a globular multi-functional protein could form microfilaments or thin filaments which both have an important function for muscle growth and development. It is a structural protein found in two forms of monomers and polymers [6]. Elzinga determined its amino acid sequence [7]. Then, in higher animals, three subtypes of actin have been found, namely α-actin, β-actin, and γ-actin. Different types of actin have different functions in muscle cell formation. In general, α-actin is involved in myocyte contraction, while β and γ-actins play a supporting role as a cytoskeleton [8,9,10]. Accumulating evidence positions actin functions in many biological processes, including participation in the process of muscle contraction, cell movement, cell division, cell signaling, the establishment and maintenance of cell connections, and cell morphology [11]. Thus, it is speculated that the actin protein is also involved in growth and development in cattle.

Actin-like 8 (ACTL8) belongs to the Actin gene family, which may play a role in the formation of the microfilament and the cytoskeleton. Our preliminary research using high-throughput sequencing of the whole bovine genome found that ACTL8 may be related to muscle growth and development (data not published). There is currently no report on the genetic variation and function of the bovine *ACTL8* gene. The ACTL8 of different species has a high degree of homology; that is to say, the structure and function of ACTL8 are similar in different species. Furthermore, ACTL8 may join to constitute the cytoskeleton for maintaining the cell morphology. ACTL8 and Rho Guanine Nucleotide Exchange Factor Family (ARHGEF) play an important role in regulating the formation of the cytoskeleton, gene transcription, cell cycle, and vesicular transport [12,13]. Cytoskeleton participates in a variety of signaling pathways, especially in the Adenosine 5’-monophosphate (AMP)-activated protein kinase (AMPK) and RhoA-ROCK signaling pathways. The analysis of GO enrichment indicated that ACTL8 may be involved in actin filament formation [14]. Actin filaments are a major component of the contractile apparatus of the skeletal muscle and the microfilaments of the cytoskeleton of eukaryotic cells [15]. Thus, it appears that ACTL8 could regulate the contractile mechanism of skeletal muscle. Muscle fiber diameter, connective tissue, density, and intramuscular fat of muscle tissue all have great effects on beef tenderness and production [16]. However, there is no research report on the *ACTL8* gene as a molecular marker candidate gene that could be used for cattle breeding. 

In this study, we aimed to identify mutations associated with bovine growth traits on the cattle *ACTL8* gene to aid in the molecular breeding of beef cattle. Therefore, we found that seven mutations of the *ACTL8* gene could act as markers in molecular breeding and selection of the growth traits of cattle.

## 2. Materials and Methods

The protocols and procedures used in this study were approved by the Faculty Animal Policy and Welfare Committee of Northwest A&F University (FAPWC-NWAFU, Protocol number, NWAFAC1008).

### 2.1. Numbers and Description of Experimental Animals

As shown in Table 1 and Supplementary Materials Figure S1, seven different Chinese cattle breeds (Qinchuan cattle, QC; Xia’nan cattle, XN; Jinnan cattle, JN; Jiaxian cattle, JX; Nanyang cattle, NY; Denan cattle, DN; Guyuan cattle, GY; total n = 1138) were chosen for the study. All cattle were healthy and female. Furthermore, the same breed was in the same feeding conditions. Blood samples were taken by venous blood collection. Then, the corresponding body size data such as withers height, body length, body weight, hucklebone width, chest depth, rump length, hip width, chest breadth, height of hip cross, abdominal girth, cannon circumference, and chest girth were recorded. The muscle, fat, heart, liver, kidney, spleen, and lung tissues were collected from Qinchuan cattle (3 months old embryo; 5 days old; 24 months old, n = 3) for the expression test.

### 2.2. DNA Isolation and Primers Design

We extracted genomic DNA from 1138 cattle using the phenol chloroform extraction method [17], and the concentration was uniformly adjusted to 25 ng /μL for building DNA pools. Based on the bovine *ACTL8* gene reference sequence in the NCBI database (GenBank accession no. AC_000159.1), seven primers (S1–S7) were designed to screen for variations in *ACTL8* (Supplementary Materials Table S1).

### 2.3. PCR Amplification and DNA Sequencing

We randomly chose fifty DNA samples of each breed, then transferred to the same tube for constructing the different breed of bovine genomic DNA pools [18]. Using a genomic DNA pool as a template for PCR amplification, PCR was performed according to the recommended system and procedure of 2× Taq PCR Master Mix (TIANGEN, Beijing, China) instruction, and the annealing temperature was set with the temperature value in Table 2. After that, the PCR product was detected by electrophoresis on 1.5% agarose gels. The single PCR product was detected by first-generation DNA sequencing at Shengong company (Shanghai, China) [19].

### 2.4. Genotyping of Mutations in the ACTL8 Gene

After DNA pool-sequencing, seven mutations (S1–S7) were identified in the *ACTL8* gene. Among them, the SNP sites of S1–S5 were analyzed using the Restriction Fragment Length Polymorphism (RFLP) technique. The S6–S7 loci were genotyped by electrophoresis on 3.5% agarose gels stained with ethidium bromide. A pair of primers (S2) were used to amplify the fragment of the SNP2 locus. Downstream primers of S4 were redesigned to detect SNP4. The new primers S1, S3, and S5 were designed to create digestion sites for genotype analysis of the PCR products (Table 2) 

The 10μL PCR amplification products of these locations were digested with 10 U of restriction enzymes (Takara, China) for 10 h at 37 °C following the supplier’s protocol. The information about the restriction enzymes is shown in Table 2. The digested products were detected by electrophoresis on 3.0% agarose gels stained with ethidium bromide.

### 2.5. Tissues Expression Profiling Test

RNA was extracted from different tissues of Qinchuan cattle by the TRIzol method. The cDNA was obtained by reverse transcription using the PrimeScript RT kit (TaKaRa, Kusatsu, Shiga Prefecture, Japan), and the concentration was controlled to a uniform 50 ng/μL.

Primers of the ACTL8 mRNA expression test were designed using Beacon Designer 8.14 software (Premier Biosoft International, Palo Alto, CA, USA), and β-actin was used as an internal reference gene. The ACTL8 mRNA quantitative primers were designed with the reference sequence of accession number XM_015462511.1 (GI: 982978130) in GenBank (Table 2). 

In this test, the qRT-PCR reaction system and conditions were derived from the qRT-PCR reaction standard provided by the SYBR^®^ Premix Ex TaqTM II (TaKaRa, Kusatsu, Shiga Prefecture, Japan) kit instruction.

### 2.6. Statistical Analysis

Genotypic and allelic frequencies of the *ACTL8* gene were calculated by EXCEL2010 software. Hardy-Weinberg equilibrium (HWE) in the different groups was evaluated through the χ2 test in the SHEsis software [20]. The population genetic parameters were obtained by Nei’s method, including the values of corresponding expected homozygosity (Exp-Hom), expected heterozygosity (Exp-He), effective allele numbers (Ae), and the polymorphism information content (PIC)[21]. The general linear model was used to analyze the association of genotypes with body size data. Considering the influence of variables, a simplified model was established by unifying the controllable variables: Yijk = μ + Ai + Gj + Eijk, where Yijk is the observation of the body size data, μ is the overall mean, Ai is the effect of age, Gj is the effect of mutations, and Eijk is the random residual error. Significance was determined by the LSD analysis in SPSS19.0 software (IBM, Armonk, NY, USA). Ninety-five percent confidence intervals were constructed for the genotypic effects. Based on the amplification efficiency of the target gene and the reference gene, according to the CT value obtained by qRT-PCR, a group close to the average value was selected as the control group. Then, the relative expression level was calculated using 2 ^−ΔΔCt^. The GraphPad Prism 5.0 software (GraphPad Software Inc., San Diego, CA, USA) was used for the analysis.

## 3. Results

### 3.1. Seven Mutations of Cattle ACTL8 Gene

**Figure 1:**
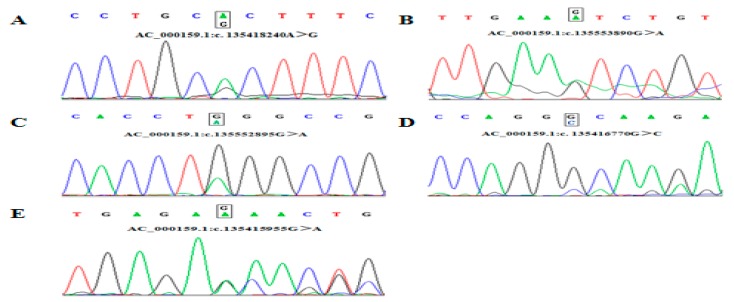
Sequencing of five SNPs in *ACTL8* gene. (**A)** SNP1 locus in 1 intron; (**B**) SNP 2 is synonymous mutation [CTG (162Leu)>CTA (162Leu)] in 10th exon; (**C**) SNP 3 locus in 10th exon; (**D**) SNP 4 locus in 5’UTR region; (**E**), SNP 5 locus in 3’UTR region.

DNA pool sequencing and Blastn alignment identified five genetic variant loci within the cattle *ACTL8* gene (AC_000159.1). SNP 1 (c. 135418240A>G) located in the first intron region, which was identified by the Hha I digestion; SNP 2 (c. 135552895G>A) and SNP 3 (c. 135553890G>A) located the 10th exon region, where SNP 2 is synonymous mutation [CTG (162Leu)>CTA (162Leu)]. SNP 2 was identified by the Mae I digestion. Similarly, SNP 3 was identified by Aha III digestion. SNP 4 (c. 135416770G>C) and SNP 5 (c.135415955A>G) located in the 5 ‘UTR and 3’ UTR region. SNP 4 was identified by the Hae III digestion. Then, SNP 5 was identified by the Asu I digestion (Figure 1, Supplementary Materials Figure S2). 

**Figure 2:**
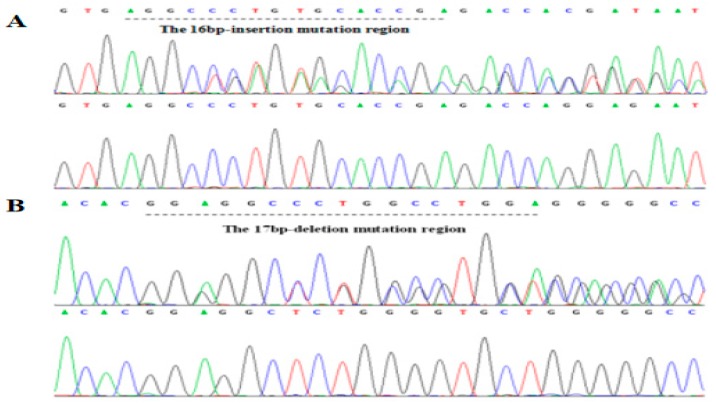
Sequencing of two indels in *ACTL8* gene. (**A**) indel 2 is 16 bp insertion on rs714529542; (**B**) indel 1 is 17 bp deletion on rs714871276.

The 17 bp deletion on rs714871276 (indel 1) and 16 bp insertion on rs714529542 (indel 2) were found in Chinese cattle by PCR and sequencing (Figure 2, Supplementary Materials Figure S2). According to the results of electrophoresis, different fragment indel types were divided into three genotypes, Mutation (Mutation-Mutation, MM), Heterozygous (wild-mutation, WM) and Wild (wild-wild, WW).

### 3.2. Genotypic and Allelic Frequencies and Genetic Diversity

The genotypic and allele frequencies and genetic diversity values are shown in Table 3 and Table 4, respectively. According to Nei’s methods, it showed gene expected homozygosity greater than 0.5 and effective allele numbers greater than 1 in the seven mutations of all cattle groups. The polymorphism information content in the population genetic parameters reflects the polymorphism of the variation in the population. The results demonstrated that mutations of the *ACTL8* gene belonged to low or moderate genetic diversity in all populations (range of PIC < 0.375, > 0.106). Mutations of the *ACTL8* gene in JX and GY cattle groups mostly belonged to low genetic diversity (PIC < 0.25) (Supplementary Materials Table S2); however, mutations were showed moderate genetic diversity in other cattle groups (0.25 < PIC < 0.50). There was a significant difference between different cattle breeds in mutations. About the HWE test, the frequency of some mutations in different cattle groups accorded with the rule of HWE (*p* > 0.05).

### 3.3. Association between Mutations of ACTL8 Gene and Growth Traits

The establishment of associations between different genotypes and growth traits was carried out in Chinese cattle breeds. 

As shown in Table 5, in the QC breed, SNP 1,2,4, and indel 2 were significantly associated with some forequarter traits (chest girth, depth or breadth; *p* < 0.05 or *p* < 0.01). Also, SNP 2,3,5, and indel 1 all had a significant association with the rump length (*p* < 0.05 or *p* < 0.01). SNP 5 and indels 1 and 2 were significantly associated with the body length or withers height (*p* < 0.05 or *p* < 0.01). 

Results showed that in XN cattle (Table 6), mutations also had significant relationships with forequarter traits (withers height or chest phenotypes) (*p* < 0.05 or *p* < 0.01). SNP 2 and 3, as well as indel 1 and 2, were significantly associated with the height of hip cross or rump length (*p* < 0.05 or *p* < 0.01). 

The results of the association analysis between mutations and growth traits in other cattle breeds were shown in Supplementary Materials Table S3. SNP1 and 2 were significantly associated with growth traits in other cattle breeds (JX, JN and NY cattle). SNP 3, indel 1, and indel 2 were significantly associated with the height of hip cross in NY cattle (*p* < 0.05 or *p* < 0.01). In GY cattle, indel 1 had a significant association with the chest girth (*p* < 0.05).

### 3.4. The Tissue Expression Profile of ACTL8 in QC Cattle

**Figure 3:**
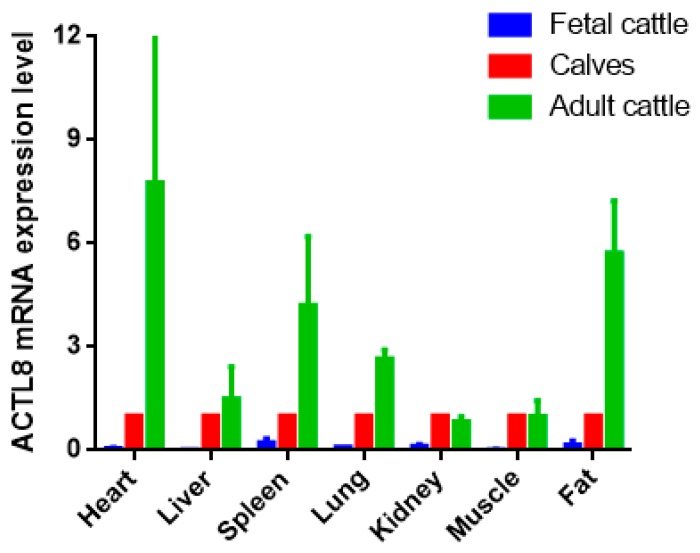
ACTL8 mRNA expression in different period of QC. The values are the averages of three samples calculated by 2^−∆∆Ct^. Error bars represent the standard error (SE) (n = 3) calculated by ∆Ct. The *β-actin* gene was chosen as the internal reference gene for the qRT-PCR.

Heart, liver, spleen, lung, kidney, muscle, and adipose tissues were utilized to detect the expression of the *ACTL8* gene. The result showed the different expression levels in each tissue at three growth stages. To reduce the effects of age and growth of fetal and adult cattle, we picked the calf growth stage as the benchmark. Then, the result revealed that ACTL8 was differentially expressed in the same tissues at distinct stages. It was indicated that the tissue expression level of the *ACTL8* gene was the highest in adult cattle and the lowest in fetal cattle. In heart, spleen, lung and adipose tissues, a significantly different expression level of ACTL8 was indicated from fetal to the adult stage. (*p* < 0.01). In kidney and muscle tissues, the expression level of the *ACTL8* gene was the highest in calves and the lowest in the fetal cattle, but the difference in calves and adult cattle stage was not statistically significant (*p* > 0.05) (Figure 3).

## 4. Discussion

Through the current reports, *ACTL8* gene may play an important role in tumorigenesis and myogenesis [22,23,24]. In this study, we aimed to find out whether the *ACTL8* gene could be used as a candidate gene to perform bovine marker-assisted selection.

Therefore, this study investigated polymorphic sites of the *ACTL8* gene in Chinese cattle. Seven mutation sites of the *ACTL8* gene were found in the non-coding region and the coding region (synonymous mutation). Mutations were tested in seven Chinese cattle populations (n = 1138) by PCR-RFLP and other methods. Based on the analysis of population genetic index and PIC value, it was indicated that polymorphic sites on the *ACTL8* gene were in high level of genetic diversity. The frequency of different SNP loci was different, which may be caused by breed factors. In this study, XN, DN and GY cattle were hybrid breeds. Then, SNPs in some cattle groups were not in the Hardy-Weinberg equilibrium (*p* < 0.01), which may be attributable to artificial selection pressure leading to the reduction of genetic diversity and the occurrence of imbalance.

At present, there is no research on the association between the polymorphism of the *ACTL8* gene and growth traits of cattle breeds, but there are some studies on the association analysis of other cattle genes and growth traits or disease. It is reported that a significant association exists between SNPs of the bovine *NUCB2* gene and the growth traits of body length, body weight and average daily gain in native Chinese cattle [25]. There is a significant correlation between the polymorphisms of the *PRNP* gene and its transcription levels [26]. Our study indicated a significant association between SNP 1 and the chest girth in QC and NY cattle. The SNP 2 was mainly affected by the allele G, which was significantly associated with the withers height and height of hip cross in XN and JN cattle. Then, SNP 3 was significantly associated with the body length of JX cattle. Besides, we found in QC cattle that SNP 4 was significantly associated with the chest girth, and SNP 5 was significantly associated with the body length, rump length, withers height, and hip width. It was mainly affected by allele G in these two loci. For indels, there was an association between indel 1 and the height of hip cross in XN and JX cattle. Meanwhile, it was significantly associated with the chest girth in GY cattle. Moreover, indel 2 was indicated to be significantly associated with height of hip cross and chest girth in XN and JX cattle as well. All results of the association analysis show mutations in the *ACTL8* gene of cattle could act as molecular markers for Chinese beef cattle breeding in some growth traits. Growth traits in cattle reflect the value of beef cattle [27]. Therefore, the significant relationship between mutations in the *ACTL8* gene and cattle growth traits will help for the development of meat production traits in beef cattle.

Expression of the *ACTL8* gene in different tissues showed its low expression in the fetal stage of QC cattle. With the development of cattle, the expression level of the *ACTL8* gene increased in different tissues. It is reported that bones and muscles develop fastest in calves [28], and the heart is the first development tissue in life [29]. Development of adipose would run through the life cycle of cattle [30,31]. Our results suggest that ACTL8 may be involved in the growth and development of bovine tissues, so as to reveal the gene plays a very important biological function.

## 5. Conclusions

Overall, we found five SNPs and two indels in the bovine *ACTL8* gene by DNA-seq. The seven identified mutations in the *ACTL8* gene could act as DNA markers for beef cattle breeding to select for increased body size. Tissue expression profile of cattle *ACTL8* gene was in flux among the three growth stages. It was highly expressed in the adult stage. For further research on the role of tissue development in cattle, the *ACTL8* gene provided some basic information. In sum, it showed that the mutations could have important reference value in beef molecular breeding and selection of the growth traits of cattle. 

## Figures and Tables

**Table 1:** The information of experiment animals.

Breeds	Number	Origin	Age
QC	394	Qinchuan cattle Varieties Breeding Center, Shaanxi Province	24–36 months
XN	213	three family prairie red bull farm, Jilin Province	24 month
JN	180	cattle farm, Wanrong county, Yuncheng city, and Wutai county, Xinzhou city, Shanxi Province	24 month
JX	82	Jia county and Baofeng county, Henan Province	24 month
NY	81	Nanyang city, Henan Province	24 month
DN	44	Goulin county, Dengzhou city, Henan Province	24 month
GY	144	Guyuan City, Ningxia Hui Autonomous Region	24 month

QC, Qinchuan cattle; XN, Xia’nan cattle; JN, Jinnan cattle; JX, Jiaxian cattle; NY, Nanyang cattle; DN, Denan cattle; GY, Guyuan cattle.

**Table 2:** Primers used for the PCR-RFLP analysis and expression test of *ACTL8* gene in cattle.

Loci	Primer Sequences (5′–3′)	Restriction Enzymes	Fragment Size (bp)	Temperature (°C)
S1	F1 ACCCTGGCTTTAGATACTGA R1 ACGGCTAGTGCGTGGGGAGC (AA) G	Hha I	297, 215, 186, 82, 29	65.0
S2	F2 GGGGCAACACCCTCTACC R2 GGGAACCACCGCTCACAG	Mae I	885, 588, 297	62.5
S3	F3 GGTGGGATGGGAGAGTTT (G) AA R3 TCCTCCTTCGTCAGCCACTC	Aha III	452, 432, 20	59.4
S4	F4 CTATCCGCCCATCCCTCT R4 TCAGTGGGCGGGTCAGGA	Hae III	235, 182, 53	63.9
S5	F5 ATTGAGGGCAAGCGAAGG R5 TGCGCCAGACAGCACAGG (T) T	Asu I	268, 247, 21	64.1
DL-ACTL8	F: ATTTGCCGACCTGACACCTT	84bp		60.0
	R: GAACGACCAGATGTGCTCCA			
DL-β-actin	F: GTCATCACCATCGGCAATGAG	84bp		60.0
	R: AATGCCGCAGGATTCCATG			

**Table 3:** Genotypic and allelic frequencies in QC and XN cattle.

Mutations	Breeds	Genotype/Genotypic Frequency			Alle /Allelic Frequency	
SNP 1	QC (394)	AA/0.633	GG/0.092	AG/0.275	A/0.771	G/0.229
	XN (213)	0.925	0.045	0.030	0.940	0.060
SNP 2	QC (394)	AA/0.122	GG/0.464	AG/0.414	A/0.329	G/0.671
	XN (213)	0.473	0.014	0.513	0.270	0.730
SNP 3	QC (394)	AA/0.168	GG/0.584	AG/0.248	A/0.292	G/0.708
	XN (213)	0.396	0.434	0.170	0.481	0.519
SNP 4	QC (394)	CC/0.159	GG/0.576	CG/0.265	C/0.291	G/0.709
SNP5	QC (394)	AA/0.389	GG/0.250	AG/0.361	A/0.569	G/0.431
indel 1	QC (394)	WW/0.555	MM/0.132	WM/0.313	W/0.711	M/0.289
	XN (213)	0.651	0.163	0.186	0.744	0.256
indel 2	QC (394)	WW/0.278	MM/0.526	WM/0.196	W/0.376	M/0.624
	XN (213)	0.337	0.419	0.244	0.459	0.541

QC, Qinchuan cattle; XN, Xia’nan cattle; JN, Jinnan cattle; JX, Jiaxian cattle; NY, Nanyang cattle; DN, Denan cattle; GY, Guyuan cattle.

**Table 4:** Diversity values in QC and XN cattle.

Mutations	Breeds	HWE	Exp-Hom	Exp-He	Ae	PIC
SNP 1	QC (394)	10.689	0.646	0.354	1.547	0.291
	XN (213)	26.318	0.888	0.112	1.126	0.106
SNP 2	QC (394)	0.959 *	0.558	0.442	1.791	0.344
	XN (213)	6.742	0.606	0.394	1.651	0.317
SNP 3	QC (394)	25.635	0.587	0.413	1.705	0.328
	XN (213)	23.079	0.501	0.499	1.997	0.375
SNP 4	QC (394)	21.877	0.587	0.413	1.703	0.328
SNP5	QC (394)	10.004	0.510	0.490	1.962	0.370
indel 1	QC (394)	12.880	0.589	0.411	1.697	0.326
	XN (213)	22.488	0.619	0.381	1.615	0.308
indel 2	QC (394)	70.736	0.531	0.469	1.883	0.359
	XN (213)	22.226	0.503	0.497	1.987	0.373

Note: *Exp-He* gene expected heterozygosity, *Exp-Hom* gene expected homozygosity, A*e* effective allele numbers, *PIC* polymorphism information content, HWE: Hardy-Weinberg equilibrium (*, *p* > 0.05).

**Table 5:** Association of mutations in the *ACTL8* gene with growth traits of QC cattle.

Loci	Growth Traits	Genotypes (Mean ± SE)		
SNP 1		AA	GG	AG
	Chest girth (cm)	180.8 ± 1.3 ^A^	167.7 ± 10.3 ^B^	181.9 ± 1.7 ^A^
SNP 2		AA	GG	AG
	Chest depth (cm)	60.9 ± 2.5 ^b^	65.2 ± 0.7 ^a^	64.3 ± 0.7 ^a^
	Rump length (cm)	42.9 ± 0.7 ^b^	44.4 ± 0.3 ^a^	44.1 ± 0.4 ^a^
SNP 3		AA	GG	AG
	Rump length (cm)	44.4 ± 0.6 ^a^	43.0 ± 0.4 ^b^	44.9 ± 0.6 ^a^
SNP 4		CC	GG	CG
	Chest girth (cm)	165.1 ± 7.1 ^B^	178.0 ± 2.4 ^AB^	181.7 ± 2.1 ^A^
SNP 5		AA	GG	AG
	Withers height (cm)	128.8 ± 0.9 ^b^	132.0 ± 1.2 ^a^	130.8 ± 0.8 ^a^
	Body length (cm)	134.6 ± 1.8 ^B^	141.5 ± 1.7 ^A^	140.1 ± 1.1 ^A^
	Rump length (cm)	43.4 ± 0.5 ^B^	44.1 ± 0.4 ^AB^	45.2 ± 0.5 ^A^
	Hip width (cm)	42.2 ± 0.6 ^b^	43.6 ± 0.5 ^ab^	43.9 ± 0.6 ^a^
indel 1		WW	MM	WM
	Withers height (cm)	129.2 ± 0.6 ^a^	131.3 ± 1.2 ^a^	128.2 ± 0.7 ^b^
	Body length (cm)	138.1 ± 0.8 ^A^	138.6 ± 2.4 ^A^	133.0 ± 1.6 ^B^
	Rump length (cm)	43.5 ± 0.3 ^B^	45.5 ± 0.7 ^A^	43.1 ± 0.4 ^B^
indel 2		WW	MM	WM
	Body length (cm)	139.5 ± 1.1 ^a^	134.0 ± 1.7 ^b^	136.4 ± 2.0 ^a^
	Chest breadth (cm)	37.2 ± 0.6 ^a^	36.5 ± 0.7 ^b^	39.3 ± 0.7 ^a^
	Hucklebone width (cm)	21.9 ± 0.5 ^a^	22.2 ± 0.4 ^a^^b^	23.7 ± 0.6 ^b^

Note: The data are expressed as least square means ± standard errors (mean ± SE). Values with different superscripts within the same row differ significantly at *p* < 0.05 (a, b, ab); *p* < 0.01 (A, B, AB). Only significant associations were shown for each of the growth traits measured.

**Table 6:** Association of mutations in the *ACTL8* gene with growth traits of XN cattle.

Loci	Growth Traits	Genotypes (Mean ± SE)		
SNP 1		AA	GG	AG
	Chest depth (cm)	18.3 ± 0.1 ^a^	18.8 ± 0.4 ^a^	16.8 ± 0.3 ^b^
SNP 2		AA	GG	AG
	Withers height (cm)	129.5 ± 3.5 ^AB^	126.6 ± 0.8 ^B^	131.1 ± 0.8 ^A^
	Height of hip cross (cm)	135.0 ± 1.0 ^B^	135.8 ± 0.8 ^B^	139.0 ± 0.8 ^A^
	Chest girth (cm)	157.0 ± 31.0 ^B^	186.0 ± 1.5 ^A^	189.2 ± 1.5 ^A^
	Rump length (cm)	444.5 ± 13.5 ^ab^	439.4 ± 8.3 ^b^	470.7 ± 11.1 ^a^
SNP 3		AA	GG	AG
	Withers height (cm)	129.9 ± 1.2 ^a^	129.7 ± 0.9 ^a^	125.1 ± 1.4 ^b^
	Height of hip cross (cm)	139.3 ± 1.0 ^a^	137.8 ± 0.9 ^ab^	134.7 ± 1.5 ^b^
	Chest girth (cm)	192.4 ± 2.4 ^b^	186.7 ± 1.9 ^a^	183.6 ± 2.3 ^a^
	Chest breadth (cm)	227.0 ± 2.8 ^A^	213.8 ± 2.8 ^B^	215.3 ± 3.0 ^B^
indel 1		WW	MM	WM
	Height of hip cross (cm)	136.4 ± 0.6 ^b^	139.7 ± 1.1 ^a^	137.6 ± 1.1 ^ab^
	Chest girth (cm)	186.2 ± 1.2 ^b^	192.1 ± 3.1 ^a^	184.7 ± 2.4 ^ab^
	Rump length (cm)	447.1 ± 8.5 ^b^	487.9 ± 18.7 ^a^	440.1 ± 10.4 ^b^
indel 2		WW	MM	WM
	Height of hip cross (cm)	137.9 ± 0.8 ^a^	135.7 ± 0.7 ^b^	138.5 ± 1.0 ^a^
	Chest girth (cm)	188.8 ± 2.0 ^a^	182.1 ± 2.1 ^b^	189.6 ± 2.0 ^a^
	Rump length (cm)	477.7 ± 13.2 ^A^	428.1 ± 7.2 ^B^	458.6 ± 14.0 ^A^

Note: The data are expressed as least square means ± standard errors (mean ± SE). Values with different superscripts within the same row differ significantly at *p* < 0.05 (a, b, ab); *p* < 0.01 (A, B, AB). Only significant associations were shown for each of the growth traits measured.

## Supplementary Materials

The following are available online at https://www.mdpi.com/2076-2615/9/12/1068/s1, Figure S1: The seven cattle breeds distribution in China, Figure S2: Seven mutations in *ACTL8* gene by agarose gel electrophoresis, Table S1: Primer sequences for loci of the *ACTL8* gene, Table S2: Genotypic and allelic frequencies and diversity analyze in other cattle, Table S3: Association analyse of growth traits and mutations of *ACTL8* gene in other cattle breeds.
