# Supplementary material for: Differential Expression of ACTL8 Gene and Association Study of Its Variations with Growth Traits in Chinese Cattle
Source: Animals (Basel). 2019 Dec 2;9(12):1068. doi: 10.3390/ani9121068 (PMC6941090; doi:10.3390/ani9121068)
Supplement: Supplementary file 1 [file animals-09-01068-s001.pdf]

Supplementary files:

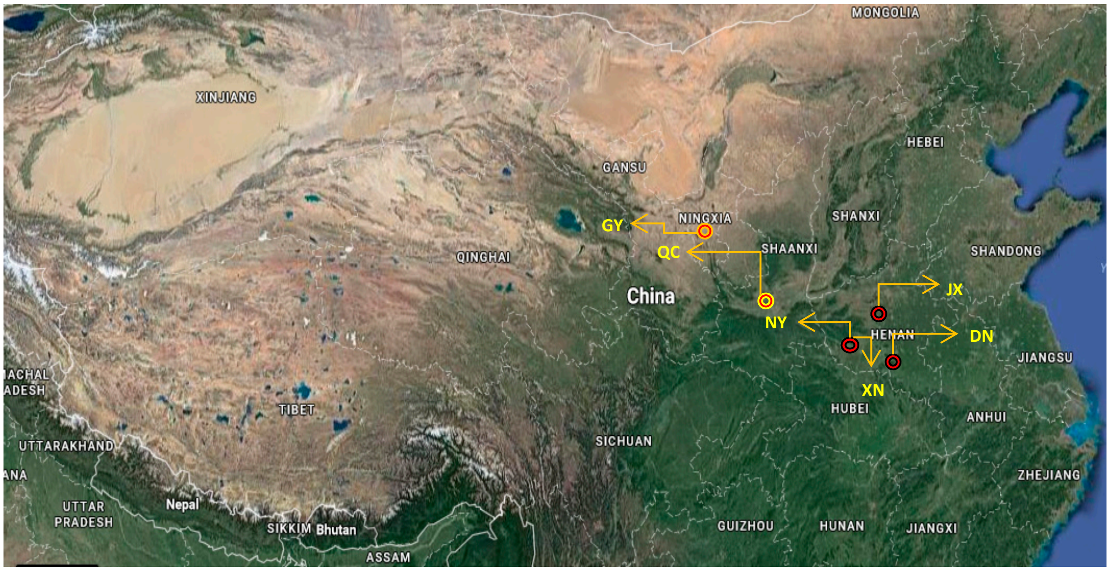

Figure S1. The seven cattle breeds distribution in China. QC: Qinchuan cattle; GY:Guyuan cattle; NY:Nanyang cattle; XN:Xianan cattle; DN:Denan cattle; JX:Jiaxian cattle

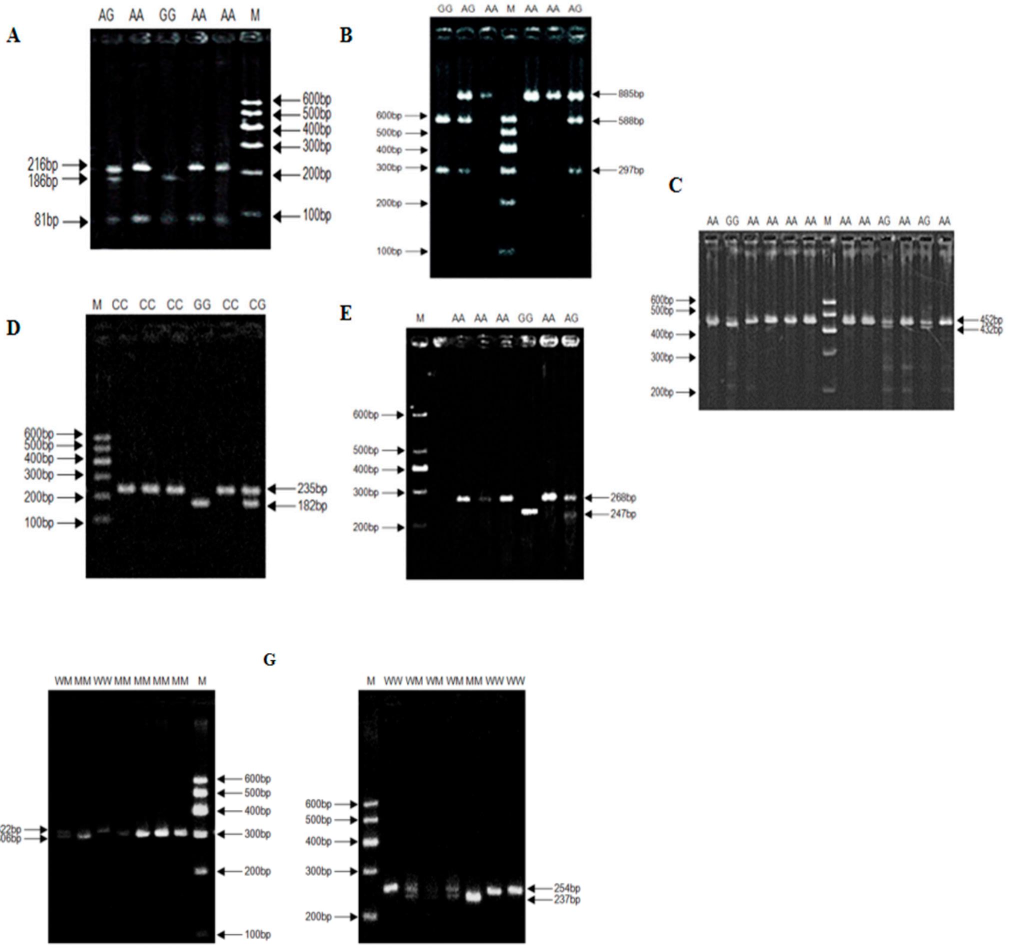

Figure S2. Seven mutations in *ACTL8* gene by agarose gel electrophoresis. A, SNP1: AA= 216 +81 bp. AG = 216 + 186 + 81 bp. GG = 186+168 bp; B, SNP 2: AA= 885 bp. AG = 885 + 588 + 297 bp. GG = 588+168 bp; C, SNP 3: AA= 452 bp. AG = 452 + 432 bp. GG = 432 bp; D, SNP 4: CC= 235 bp. CG = 235 + 182 bp. GG = 182 bp; E, SNP 5: AA= 268 bp. AG = 268 + 247 bp. GG = 247 bp; F, indel 2: WW= 306 bp. WM = 322 + 306 bp. MM =322 bp; G, indel 1: WW= 254 bp. WM = 237 + 254 bp. MM =237 bp;.

Table S1. Primer sequences for loci of the *ACTL8* gene.

Loci	Primer Sequences (5'-3')	Size (bp)	Temperature (°C)	Region
S1	F ACCCTGGCTTTAGATACTGA<br>R CACAAGGGAAGTCCAAGAA	529	64.1	Exon 1+Part of Intron 1
S2	F CCTGGCTCTTCCTGATCCTC<br>R TCCTCCTTCGTCAGCCACTC	885	62.5	Part of Exon 10
S3	F GGGGCAACACCCTCTACC<br>R GGGAACCACCGCTCACAG	848	65.0	Part of Exon 10
S4	F CTATCCGCCCATCCCTCT<br>R CCCAAGCAAACCCTACCTAC	809	62.5	Part of 5′UTR
S5	F ATTGAGGGCAAGCGAAGG<br>R AGAGGGATGGGCGGATAG	923	62.5	Part of 3′UTR
S6	F CATTACGACTGACGACCACGA<br>R GTTTGGGAGCCCCGTGACTA	254	68.0	Deletion
S7	F TCTCAGGGGCAAATGCTGA<br>R TGGGAGTTGCTGTGTTCTGA	306	64.5	Insertion

Table S2. Genotypic and allelic frequencies and diversity analyze in other cattle.

Mutations	Breeds	Genotypic frequency			Allelic frequency		HWE	Exp-Hom	Exp-He	Ae	PIC
SNP 1		AA	GG	AG	A	G					
	JN(180)	0.524	0.243	0.233	0.641	0.359	25.121	0.540	0.460	1.853	0.354
	JX(82)	0.720	0.049	0.231	0.835	0.165	1.492*	0.725	0.275	1.379	0.237
	NY(81)	0.555	0.093	0.352	0.731	0.269	0.454*	0.607	0.393	1.647	0.316
	DN(44)	0.651	0.070	0.279	0.791	0.209	0.740*	0.669	0.331	1.495	0.276
	GY(144)	0.875	0.014	0.111	0.931	0.069	2.869*	0.872	0.128	1.147	0.120
SNP 2		AA	GG	AG	A	G					
	JN(180)	0.044	0.565	0.391	0.239	0.761	0.522*	0.636	0.364	1.572	0.298
	JX(82)	0.066	0.508	0.426	0.279	0.721	0.410*	0.598	0.402	1.672	0.321
	NY(81)	0.125	0.688	0.187	0.219	0.781	2.371*	0.658	0.342	1.519	0.283
	DN(44)	0.080	0.520	0.400	0.280	0.720	0.158*	0.597	0.403	1.676	0.322
	GY(144)	0.013	0.799	0.188	0.107	0.893	0.849*	0.809	0.191	1.236	0.173
SNP 3		AA	GG	AG	A	G					
	JX(82)	0.031	0.846	0.123	0.092	0.908	2.690*	0.832	0.168	1.201	0.154
	DN(44)	0.182	0.682	0.136	0.250	0.750	16.449	0.625	0.375	1.600	0.305
	GY(144)	0.034	0.875	0.091	0.080	0.920	21.357	0.853	0.147	1.172	0.136
SNP 4		CC	GG	CG	C	G					
	GY(144)	0.042	0.868	0.09	0.087	0.913	26.589	0.841	0.159	1.189	0.146
SNP5		AA	GG	AG	A	G					
	GY(144)	0.222	0.077	0.701	0.573	0.427	27.033	0.511	0.489	1.957	0.369
indel 1		WW	MM	WM	W	M					
	JX(82)	0.164	0.509	0.327	0.327	0.673	3.293*	0.560	0.440	1.787	0.343
	DN(44)	0.435	0.087	0.478	0.674	0.326	0.466*	0.560	0.440	1.784	0.343
	GY(144)	0.625	0.118	0.257	0.754	0.246	13.733	0.629	0.371	1.590	0.302
indel 2		WW	MM	WM	W	M					
	JX(82)	0.236	0.327	0.436	0.455	0.545	0.792*	0.504	0.496	1.984	0.373
	DN(44)	0.250	0.600	0.150	0.325	0.675	7.811	0.561	0.439	1.782	0.342
	GY(144)	0.861	0.042	0.097	0.910	0.09	24.117	0.836	0.164	1.196	0.151

Note: *Exp-He* gene expected heterozygosity, *Exp-Hom* gene expected homozygosity, *Ae* effective allele numbers, *PIC* polymorphism information content, HWE: Hardy-Weinberg equilibrium (*, $p > 0.05$)

Table S3. Association analysis of growth traits and mutations of *ACTL8* gene in other cattle breeds.

Breeds	Loci	Growth traits	Genotypes（mean±SE）		
			AA	GG	AG
JX	SNP 1	Height of hip cross (cm)	128.255±0.710 ^{B}	118.500±1.658 ^{A}	123.250±1.544 ^{A}
		Abdominal girth (cm)	197.170±2.463 ^{B}	221.000±0.408 ^{A}	188.250±0.901 ^{B}
		Cannon circumference (cm)	18.160±0.153 ^{a}	17.500±0.500 ^{ab}	17.188±0.162 ^{b}

	SNP 2		AA	GG	AG
		Body length (cm)	150.250±3.881 ^{AB}	156.194±1.764 ^{A}	149.615±1.851 ^{B}
	SNP 3		AA	GG	AG
		Height of hip cross (cm)	128.500±4.500 ^{A}	122.255±0.435 ^{B}	127.417±1.519 ^{A}
		Body length (cm)	145.500±14.500 ^{ab}	154.979±1.279 ^{a}	146.333±1.874 ^{b}
	indel 1		WW	MM	WM
		Height of hip cross (cm)	130.045±1.249 ^{a}	124.857±2.219 ^{b}	127.220±0.947 ^{ab}
	indel 2		WW	MM	WM
		Height of hip cross (cm)	126.385±1.504 ^{b}	130.750±1.453 ^{a}	127.354±0.977 ^{ab}
		Chest girth (cm)	171.846±2.803 ^{ab}	177.750±3.111 ^{a}	170.708±1.82 ^{b}
JN	SNP 1	Withers height (cm)	128.815±1.031 ^{ab}	130.652±1.036 ^{a}	125.545±1.353 ^{b}
		Rump length (cm)	47.660±0.753 ^{ab}	49.762±0.831 ^{a}	46.000±1.541 ^{b}
	SNP 2		AA	GG	AG
		Withers height (cm)	131.000±2.972 ^{ab}	131.556±0.796 ^{a}	128.944±0.870 ^{b}
		Height of hip cross (cm)	134.000±2.160 ^{AB}	135.356±1.016 ^{A}	130.833±0.995 ^{B}
		Chest girth (cm)	185.000±8.297 ^{ab}	190.644±1.509 ^{a}	184.222±1.902 ^{b}
NY	SNP 1		AA	GG	AG
		Body weight (kg)	365.037±5.726 ^{b}	410.800±28.052 ^{a}	367.389±9.858 ^{b}
		Chest girth (cm)	168.519±1.751 ^{b}	179.000±5.848 ^{a}	170.472±2.102 ^{b}
		Hucklebone width (cm)	24.815±0.434 ^{B}	27.600±0.812 ^{A}	25.889±0.449 ^{AB}
	SNP 2		AA	GG	AG
		Withers height (cm)	131.500±0.500 ^{A}	125.500±0.681 ^{B}	128.333±1.667 ^{AB}
		Body length (cm)	139.000±1.000 ^{b}	141.625±1.362 ^{ab}	146.333±0.882 ^{a}
GY	indel 1		WW	MM	WM
		Chest girth (cm)	173.732±0.980 ^{b}	186.000±6.245 ^{a}	174.586±2.581 ^{b}

Note: The data are expressed as least square means ± standard errors (mean ± SE). Values with different superscripts within the same row differ significantly at $P < 0.05$ (a, b, ab); $P < 0.01$ (A, B, AB). Only significant associations were shown for each of the growth traits measured.
